# Cerebrospinal fluid cytokine levels are associated with macrophage infiltration into tumor tissues of glioma patients

**DOI:** 10.1186/s12885-021-08825-1

**Published:** 2021-10-15

**Authors:** Constanze L. Kemmerer, Jens Schittenhelm, Evelyn Dubois, Laura Neumann, Lisa M. Häsler, Marius Lambert, Mirjam Renovanz, Stephan A. Kaeser, Ghazaleh Tabatabai, Ulf Ziemann, Ulrike Naumann, Markus C. Kowarik

**Affiliations:** 1 Department of Vascular Neurology, Hertie-Institute for Clinical Brain Research, Eberhard-Karls University Tübingen, Otfried-Müller-Straße 27, Tübingen, Germany; 2 Department of Pathology and Neuropathology, University Hospital Tübingen, Calwerstr. 3, Tübingen, Germany; 3 Center for Neuro-Oncology, Comprehensive Cancer Center Tuebingen-Stuttgart, University Hospital of Tuebingen, Eberhard Karls University of Tuebingen, Tübingen, Germany; 4 German Cancer Consortium (DKTK), DKFZ partner site Tübingen, Eberhard Karls University Tübingen, Tübingen, Germany; 5 Department of Cellular Neurology, Hertie Institute for Clinical Brain Research, Eberhard-Karls University of Tübingen, Otfried-Müller-Straße 27, Tübingen, Germany; 6 German Center for Neurodegenerative Diseases (DZNE), Otfried-Müller-Straße 23, Tübingen, Germany; 7 Department of Neurology and Interdisciplinary Neuro-Oncology, Hertie Institute for Clinical Brain Research, Eberhard-Karls University of Tübingen, Otfried-Müller-Straße 27, Tübingen, Germany; 8 Department of Neurosurgery, University Hospital of Tuebingen, Eberhard Karls University of Tuebingen, Hoppe-Seyler-Str. 3, 72076 Tübingen, Germany; 9 Department of Neurology & Stroke, Eberhard-Karls University Tübingen, Tübingen, Germany; 10 Department of Neurology, Klinikum rechts der Isar, Technische Universität München, Ismaninger Str. 22, Munich, Germany

**Keywords:** B cells, Tumor associated macrophages, CD68, CD163, Cytokines, Immunohistochemistry

## Abstract

**Background:**

Diffuse gliomas are the most common malignant tumors of the central nervous system with poor treatment efficacy. Infiltration of immune cells into tumors during immunosurveillance is observed in multiple tumor entities and often associated with a favorable outcome. The aim of this study was to evaluate the infiltration of immune cells in gliomas and their association with cerebrospinal fluid (CSF) cytokine concentrations.

**Methods:**

We applied immunohistochemistry in tumor tissue sections of 18 high-grade glioma (HGG) patients (4 anaplastic astrocytoma, IDH-wildtype WHO-III; 14 glioblastomas (GBM), IDH-wildtype WHO-IV) in order to assess and quantify leucocytes (CD45) and macrophages (CD68, CD163) within the tumor core, infiltration zone and perivascular spaces. In addition, we quantified the concentrations of 30 cytokines in the same patients’ CSF and in 14 non-inflammatory controls.

**Results:**

We observed a significantly higher percentage of CD68^+^ macrophages (21–27%) in all examined tumor areas when compared to CD45^+^ leucocytes (ca. 3–7%); CD163^+^ cell infiltration was between 5 and 15%. Compared to the tumor core, significantly more macrophages and leucocytes were detectable within the perivascular area. The brain parenchyma showing a lower tumor cell density seems to be less infiltrated by macrophages. Interleukin (IL)-7 was significantly downregulated in CSF of GBM patients compared to controls. Additionally, CD68^+^ macrophage infiltrates showed significant correlations with the expression of eotaxin, interferon-γ, IL-1β, IL-2, IL-10, IL-13, IL-16 and vascular endothelial growth factor.

**Conclusions:**

Our findings suggest that the infiltration of lymphocytes is generally low in HGG, and does not correlate with cytokine concentrations in the CSF. In contrast, macrophage infiltrates in HGG are associated with CSF cytokine changes that possibly shape the tumor microenvironment. Although results point towards an escape from immunosurveillance or even exploitation of immune cells by HGG, further studies are necessary to decipher the exact role of the immune system in these tumors.

**Supplementary Information:**

The online version contains supplementary material available at 10.1186/s12885-021-08825-1.

## Background

Glioblastoma (GBM) is the most common type of malignant tumor in the central nervous system of adults with an unfavorable prognosis [1]. The median survival of GBM patients, even in clinical trial populations with multimodal therapies including irradiation, temozolomide-based chemotherapy and tumor-treating fields is still in the range of 1.5 years [2]. More effective treatments are thus urgently needed. In this regard, immunotherapy-based strategies have moved into focus as effective new treatment options [3].

It has been shown that the immune system is generally capable of responding to cancer cells [4]. In a variety of tumor types the development of tertiary lymphoid structures and immune cell accumulations resembling lymph nodes has been associated with positive outcomes [5, 6]. Regarding gliomas, there is evidence that tumor infiltrating effector T cells positively affect the survival time, however, these studies show partially inconsistent results [7–10]. Recent studies using anti-tumor vaccines in glioblastoma, could show sustained T cell responses in phase I clinical trials, but the prognosis remains almost uniformly fatal [11, 12]. Immune escape by glioblastoma still needs to be overcome for effective application [13].

The involvement of B cells in anti-tumor immunity has rarely been studied in gliomas so far and, when reported, B cell percentages in the tumor tissue were low [9, 14, 15]. In contrast, Candolfi et al. (2011) could show that B cells play a critical role as antigen presenting cells in T cell mediated anti-tumor immunity as evidenced by the absence of GBM specific T cell precursors in B cell deficient mice [16]. Moreover, oligoclonal bands have been detected in the CSF of glioma patients [17].

Additionally, various cytokines in the CSF such as tumor necrosis factor-α (TNF-α), transforming growth factor-β (TGF-β), interferons, interleukins (IL) 2, 4, 6, 8 10, 12 and 13, hypoxia-inducible factors (HIF), granulocyte–macrophage colony-stimulating factor (GM-CSF) and vascular endothelial growth factor (VEGF) seem to play a role in the regulation of the glioma microenvironment [18]. Further, it has been shown that cytokine concentrations in the CSF are associated with the infiltration rate of immune cells such as tumor associated macrophages (TAM) [19]. This points towards an interaction of immune cells and cytokines between the tumor tissue and the CSF compartment.

In tumor immunity, TAMs have been attributed a key role in shaping the tumor microenvironment [20]. Infiltrating monocytes may differentiate into tissue macrophages and further polarize towards a pro-inflammatory M1 or anti-inflammatory but pro-tumorigenic M2 phenotype [21]. While CD68 has previously been used as a more universal macrophage marker, CD163 is associated with the M2 phenotype [22–24]. Moreover, the depletion of microglia reduces glioma progression, emphasizing the additional importance of tumor-microglia interactions [25].

The aim of this study was to systematically evaluate the infiltration of CD45^+^ leucocytes, CD68^+^ macrophages and CD163^+^ M2 type macrophages into defined HGG tissue sections by immunohistochemistry (IHC) and to investigate the correlation of these immune cell subtypes with cytokines in the CSF.

## Methods

### Standard protocol approvals and patients

Patients were chosen according to the following criteria IDH^WT^ GBM (WHO grade IV) or IDH^WT^ AA (WHO grade III); ii) availability of CSF and tumor tissue before initiation of radio- and chemotherapy and iii) signed informed consent permitting usage of biological samples for research purposes. After approval, material of 18 patients matching these criteria was kindly provided by the biobank of the Center for Neuropathology and the biobank of the Hertie Institute for Clinical Brain Research (HIH) in Tübingen. Additionally, CSF samples of age- and gender-matched controls for the GBM patients (4 with cognitive deficits, 5 with affective disorder, 2 with normal pressure hydrocephalus, 1 with migraine and 2 healthy controls) were kindly provided by the biobank of the HIH in Tübingen and were assessed for cytokine concentrations. Inflammatory processes among the control patients were unlikely considering the CSF parameters.

### Tumor tissue handling

For immunohistochemistry, paraffin embedded tumor tissue was sliced (4 μm), processed in line with the routine diagnostic workup at the Institute for Neuropathology in Tübingen and controlled on hematoxylin/eosin HE stains for sufficient tumor content. Immunohistochemical (IHC) staining was carried out on an autostainer using the Ventana Benchmark IHC optiView System (Roche, Ludwigsburg, Germany). The antibodies employed were mouse anti human CD68 (Dako, Glostrup, Denmark, PGM-1, 1:1200) and mouse anti human CD163 (AbD Serotec, Oxford, UK, cloneEDHu-1, 1:1000) for macrophage detection and mouse anti human CD45 (Dako, Glostrup, Denmark, clone 2B11, 1:1200) for the assessment of blood derived leucocyte infiltration in general, as a prescreening for lymphocytes including B cell infiltration. As a positive control for immune cells human tonsil was stained.

For O6-Methylguanin-DNA-Methyltransferase (MGMT) pyrosequencing, a total of 250 ng of genomic DNA isolated from tumor tissue (BlackPREP FFPE kit, Analytik Jena, Germany) was subjected to bisulfite conversion, using the „Promega MethylEdge Bisulfid Conversion System (Promega, Madison, WI, USA) according to the manufacturer’s protocol. PCR amplification and pyrosequencing covering the five CpG islands 74–78 in exon 1 of the MGMT locus on chromosome 10q26 were performed with the PyroMark Q24 CpG MGMT kit (Qiagen, Hildesheim, Germany). The mean percentage of methylated alleles at all five CpG loci was used for the analysis and established cutoffs (< 8% versus > 8% methylation level) for classification as methylated versus unmethylated were applied [26].

### Cytokine measurements in CSF

In order to measure CSF cytokine concentrations, the V-PLEX Human Cytokine 30-Plex Kit (Meso Scale Diagnostics, Rockville, MD, USA) and the Human CXCL13/BLC/BCA-1 DuoSet ELISA (R&D Systems, Abington, UK) were used to determine cytokine concentrations in the CSF according to the manufacturers’ protocols. The Meso Scale cytokine assay was run on the Sector Imager 6000 (Meso Scale Diagnostics, Rockville, MD, USA). The CXCL13 ELISA was measured on the Sunrise Reader (TECAN, Crailsheim, Germany). Two replicates were measured in case enough CSF was available. For the remaining samples single measurements where used for analysis (see supplements Table 1 for details). Further CSF parameters were retrieved from the SAP system of the University Hospital Tübingen.

### IHC data analysis

**Fig. 1:**
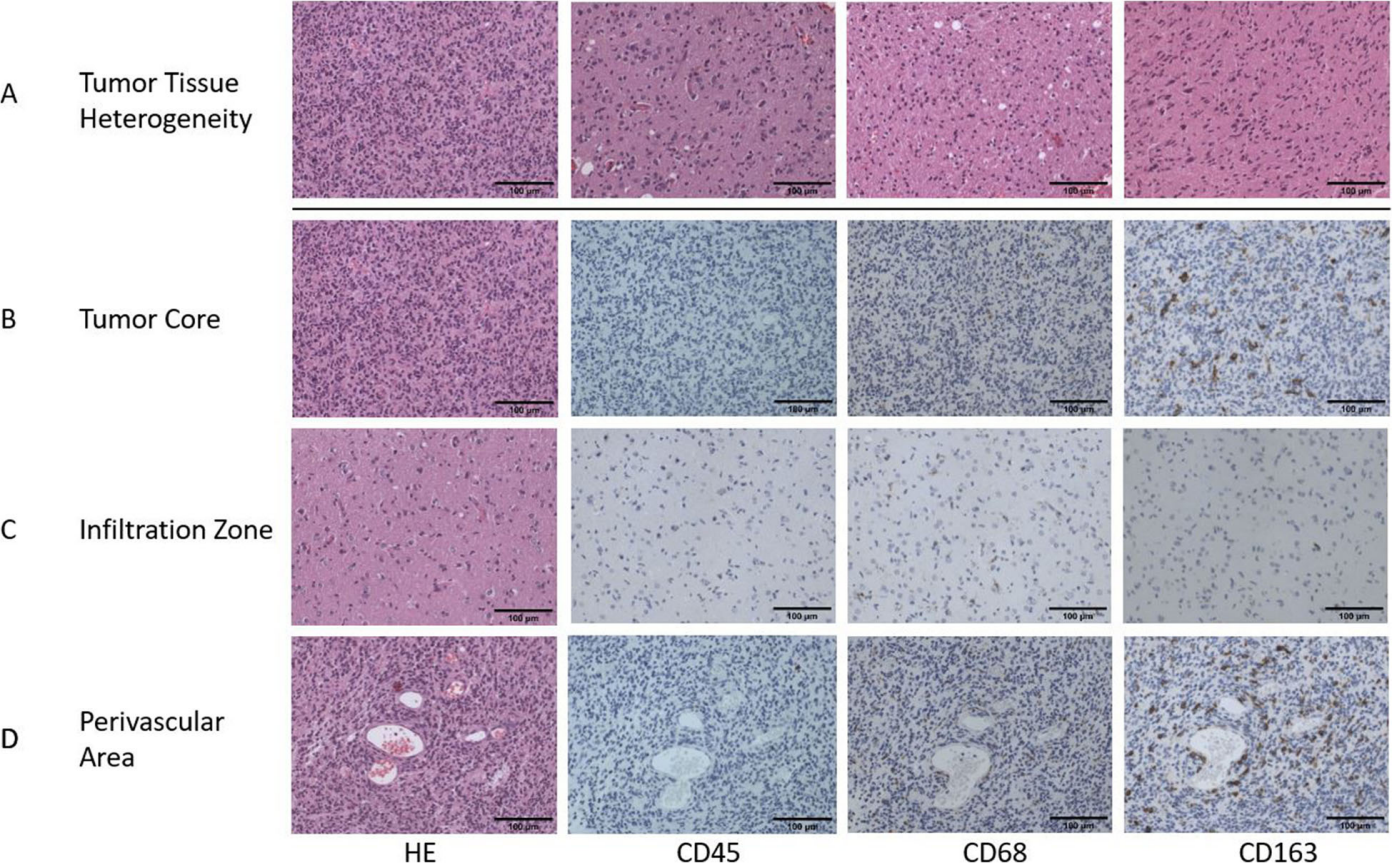
Immunohistochemistry. Representative microscopic images (200x magnification, scale bar 100 μm) of one GBM patient including the HE (first column) and the IHC staining for CD45 (second column), CD68 (third column) and CD163 (fourth column) Images demonstrate the morphological heterogeneity within the tumor tissue (**A**), the tumor core (**B**), the infiltration zone (**C**) and the perivascular area (**D**)

Photomicrographs (339 × 457.2 μm) of representative tumor sections were taken at 200x magnification under a light microscope using a Progres C10 camera system (JenOptik, Germany) in the same region of the tumor slide for every staining. For homogeneous tumors a minimum of three different areas of the tissue were used for analysis. For heterogeneous tumors, at least one image of each morphologically different area (a minimum of three regions in total) was used for analysis. We did this to reduce a possible sampling bias since we wanted to cover the full diversity of the tumor and not just selective areas of homogeneous tissue. Pictures were further rated as “core tumor” or “infiltration zone” into pre-existing tissue by a board-certified neuropathologist. Additionally, for each tumor the surrounding 50 μm of at least two large vessels (> 5 μm) were analyzed and determined as perivascular region (Fig. 1). CD68^+^, CD163^+^ and CD45^+^ cells with distinct cytoplasmic staining were counted manually using QuPath Software Version 0.2.1 (University of Edinburgh, UK). Counts were further analyzed as percentage of stained cells relative to overall stained nuclei and absolute stained cells per mm^2^ for core tumor, perivascular area and infiltration zone.

### Statistics

Data analysis for cytokine measurements were assessed using MSD Discovery Workbench software 3.0 (Meso Scale Diagnostics, Rockville, MD, USA). Statistical analyses were conducted using SPSS (IBM SPSS Statistics Version 25.0 for Windows, IBM Corportion, Armonk, NY, USA). In addition, graphs were designed using GraphPad Prism (Version 8 for Windows, GraphPad Software, San Diego, California USA). After significant deviations from normality were detected in the sample subgroups and variables (Kolmogorov-Smirnoff-test with *p* < 0.05), nonparametric tests were applied on the data.

For the IHC data, we tested for differences in immune cell staining (relative and absolute immune cell infiltration) between AA and GBM for the tumor core and perivascular area using the Mann-Whitney-U test. Further, differences between the tumor core and perivascular area were assessed using the Wilcoxon rank test. Accordingly, differences between the tumor core and infiltration zone were analyzed if at least one infiltration zone was present in the sample. Lastly, differences between the infiltrates (CD45^+^, CD68^+^ and CD163^+^ cells) were assessed by the Kruskal-Wallis-Test.

Concerning the cytokines, data cleaning was conducted as follows: Values exceeding the the upper limit of detection (ULoD) were set to the highest standard, while values below the lower limit of detection (LLoD) were set to zero. When available, the mean of the duplicates was calculated and used in further analyses (see supplementary Table 1 for details). Since measures of several cytokines in the AA group were below the detection limit and the matched control group was chosen according to the GBM patient criteria we excluded the analysis between AA and controls for the single cytokine analyses. We further excluded variables with less than 5 values per group above the LLoD for the comparisons concerning GBM which included CXCL-13, GM-CSF, IL-17, IL-1α, TNF-β and IL-4. We compared the remaining CSF cytokine concentrations between GBM patients and controls using the Mann-Whitney-U test. To determine common underlying pattern of the cytokines, a principal component analysis (PCA) was conducted using data of AA, GBM and control samples. Due to low variance, CXCL-13, GM-CSF, IL-17, IL-1α and TNF-β were excluded from the analysis. Further, MCP-1, IL-5, IL-7 and VEGF were removed due to low sampling adequacy (Kaiser-Meyer-Olkin, KMO, value < 0.5). Regression factor scores were compared between GBM, AA and controls using the Kruskal-Wallis-Test. Next, for the GBM subgroup immune cell staining was correlated with the single cytokine concentrations and with the factor scores retrieved from the PCA. Further, we assessed whether cytokine concentrations and immune cell staining in the GBM patients differed between MGMT methylation status and whether immune cell staining differed between the sexes using the Mann-Whitney-U test. Lastly, correlations of immune cell infiltration and patient age or overall survival (defined as days until death or if unknown until last entry in the SAP system at the time of data retrieval) were examined. In line with previous analyses, CXCL-13, GM-CSF, IL-17, IL-1α, TNF-β and IL-4 were excluded from these contrasts because of less than 5 values above detection limit per group.

For this exploratory analysis, the *p*-value for statistical significance was set to *p* < 0.01 for each test to find potentially relevant effects.

## Results

### Sample characteristics

For patients in the GBM group, the mean age was 63 years (range: 31–79), mean overall survival time was 386 days, 5 patients were female (36%) and 9 patients were male (64%) and the distribution of MGMT promotor methylation status was 50% unmethylated and methylated. 50% of the GBM patients showed a blood brain barrier (BBB) disruption and in two patients, oligoclonal bands type 2 were detected in the CSF. For the AA group the mean age was 63 years (range: 45–85), the mean overall survival was 183 days at time of analysis, 2 patients were male (50%) and female (50%) each and the MGMT promotor methylation status was unmethylated in 75% and methylated in 25%. Two patients showed a disruption of the BBB. The control group had a mean age of 63 years, 36% of the participants were women and 64% men. Detailed patient and control group characteristics are shown in Tables 1 and 2, respectively.

**Table 1:** Patient characteristics

Patient	Sex	Disease	MGMT status	Overall survival	Number of leucocytes	IgG Index	Albumin quotient	BBB disturbance	OCB
1	f	GBM	M	559	6	3.16	4.4	no	2
2	f	GBM	M	49	1	0.36	7.4	no	1
3	m	GBM	M	1387	1	0.62	20.9	yes	1
4	f	GBM	M	335	3	0.54	18.8	yes	1
5	m	GBM	U	126	1	0.44	10.4	yes	N.A
6	f	GBM	U	308	1	0.44	2.5	no	1
7	m	GBM	U	522	4	0.78	44.8	yes	4
8	m	GBM	U	689	1	0.46	2.6	no	N.A
9	m	GBM	U	24	3	0.52	12.8	yes	1
10	m	AA	U	38	1	0.48	22.5	yes	4
11	m	GBM	U	323	6	0.52	11.6	yes	1
12	m	GBM	M	108	1	0.5	5.8	no	1
13	f	GBM	M	96	2	0.51	6.7	no	4
14	m	AA	U	600	4	0.47	5.9	no	1
15	f	AA	U	79	1	0.49	8.3	yes	N.A
16	f	AA	M	14	1	0.46	5.0	no	1
17	m	GBM	U	13	6	0.43	8.0	no	5
18	m	GBM	M	867	1	0.52	10.7	yes	1

Table 1*:* Demographic and cerebrospinal fluid characteristics (CSF) of the HHG patiens. Overall survival is presented in days and number of leukocytes is presented in cells per μl CSF. All HGG are isocitrat-dehydrogenase wildtype gliomas. *Abbreviations*: *f* female, *m* male, *MGMT* O6-Methylguanin-DNA-Methyltransferase, *IgG Index* Immunoglobulin G index, *BBB* Blood brain barrier, *OCB* Oligoclonal bands with numbers referring to OCB type: 1: no OCB visible in serum and CSF, 2: OCB only visible in CSF and not in serum, 3: OCB exclusive to CSF and additionally identical OCB in serum and CSF, 4: identical OCB in CSF and serum, 5: paraprotein split into closely adjacent bands, *GBM* Glioblastoma, *AA* Astrocytoma grade III, *M* Methylated MGMT promotor, *U* Unmethylated MGMT promotor, *N.A.* Not available

**Table 2:** Characteristics of control subjects

Patient	Sex	Disease	Number of leucocytes	IgG Index	Albumin quotient	BBB disturbance	OCB
19	m	Healthy	3	N.A.	N.A.	N.A.	N.A
20	m	Cognitive deficit	2	2.3	4.8	no	N.A
21	f	NPH	N.A.	N.A.	N.A.	N.A.	N.A
22	m	Cognitive deficit	1	0.45	6.9	no	N.A
23	f	NPH	1	0.47	5.9	no	N.A
24	m	Healthy	1	0.51	5.5	no	N.A
25	m	Bipolar disorder	1	0.47	5.7	no	N.A
26	f	Cognitive deficit, Depression	5	0.5	14.5	Yes	N.A
27	f	Migraine	2	0.45	8.2	yes	N.A
28	m	NPH	1	0.41	4.6	no	N.A
29	m	Bipolar disorder	1	0.52	7.1	no	N.A
30	m	Episode of Depression	1	0.51	4.9	no	N.A
31	m	Depression	< 1	0.39	13.3	no	N.A
32	f	Cognitive deficit	1	0.50	7.2	no	N.A

Table 2*:* Demographic and cerebrospinal fluid characteristics (CSF) of the control group. Number of leukocytes is presented in cells per μl CSF. *Abbreviations*: *f* female, *m* male, *NPH* Normal pressure hydrocephalus, *MGMT* O6-Methylguanin-DNA-Methyltransferase, *IgG Index* Immunoglobulin G index, *BBB* Blood brain barrier, *OCB* Oligoclonal bands, *N.A.* Not available

### Difference in immune cell distributions between the different tumor areas

**Fig. 2:**
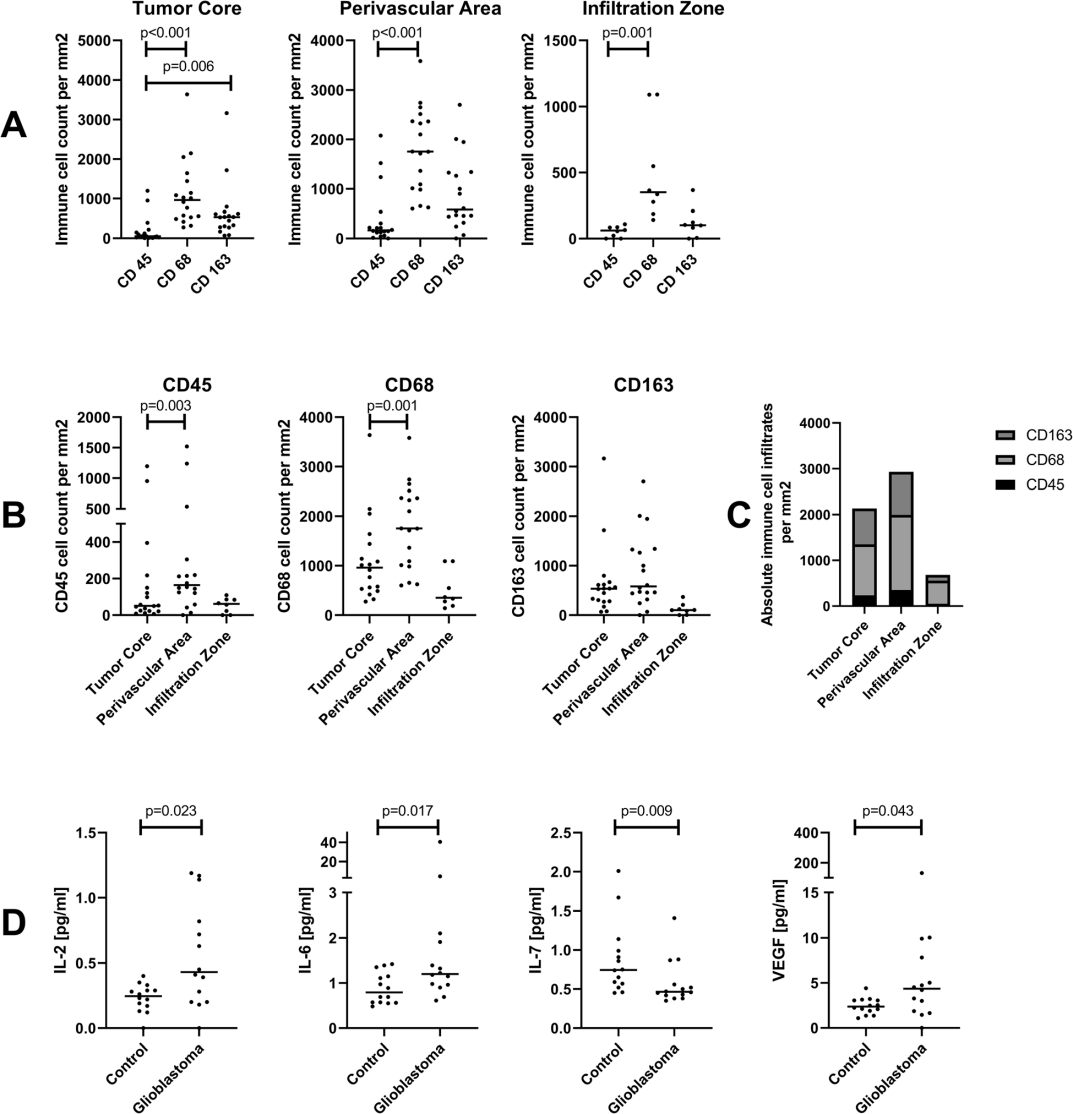
Differences in immune cell infiltrations and cytokine concentrations. **A** Differences in the infiltration of CD45^+^, CD68^+^ and CD163^+^ cells (median cell counts per mm^2^) within each tumor area. **B** Differences in the immune cell infiltrates (median cell counts per mm^2^) between the tumor areas. **C** Overview on the distribution of absolute immune cell infiltrates (median cell counts per mm^2^) for the different tumor areas. **D** Median concentrations (in pg/ml) of IL-2, IL-6, IL-7 and VEGF in the CSF of GBM patients vs. controls as assessed by the multiplex assay

We analyzed the absolute count and percentage of CD45^+^ leucocyte, CD68^+^ macrophage and CD163^+^ M2 type macrophages in the tumor core, perivascular area and tumor infiltration zone (Figs. 1 and 2). There were no significant differences between AA and GBM except from a trend towards higher CD163^+^ macrophage infiltration in GBM for absolute and relative infiltration. Therefore, we continued the analysis with AA and GBM combined as HGG. Within the different tumor areas, we observed a significant lower count of CD45^+^ leucocytes when compared to CD68^+^ macrophages in all three examined areas and a significantly lower count of CD45^+^ leucocytes compared to CD163^+^ M2 type macrophages (Fig. 2A/C). When analyzing the cell types between the different tumor areas, infiltration of CD45^+^ as well as CD68^+^ cells were significantly higher in perivascular areas compared to the tumor core (Fig. 2B/C). Concerning the difference in immune cell infiltration between the infiltration zone and tumor core, we found a tendency towards higher absolute macrophage infiltration (CD68^+^ as well as CD163^+^) for the tumor core which did not reach statistical significance on a *p* < 0.01 level (*p* = 0.017 for CD68^+^ and *p* = 0.012 for CD163^+^, Fig. 2B/C).

Similar results were obtained when looking at the percentage distribution of the different cells in the examined tumor areas. The percentage of CD45^+^ cells (tumor core: median 1%, mean: 4%; perivascular area: median: 3%, mean: 7%; tumor infiltration zone: median: 3%, mean: 3%) was significantly lower than the percentage of CD68^+^ cells (tumor core: median 19%, mean: 22%%; perivascular area: median: 27%, mean: 27%; tumor infiltration zone: median: 19%, mean: 21%) within the tumor core (*p* < 0.01), perivascular area (p < 0.01) and tumor infiltrating zone (*p* = 0.01). Moreover, in the tumor core the percentage of CD163^+^ cells (median: 9%, mean: 12%) was significantly higher than the percentage of CD45^+^ cells (*p* = 0.006). When examining the percentage distribution of infiltrating cells between the different tumor areas, we observed significantly higher percentages of CD45^+^ cells in the perivascular area compared to the tumor core (*p* = 0.008). The increased percentage of CD68^+^ cells in the perivascular area in contrast to the tumor core failed to reach statistical significance (*p* = 0.022).

### Differences in cytokine concentrations and principal component analysis

We next analyzed the concentrations of 30 cytokines harboring inflammatory and chemotactic properties in the corresponding CSF samples of the tumor patients and matched controls (Fig. 2D). The concentration of IL-7 was significantly lower in GBM patients compared to controls (U = 41, *p* = 0.009). Additionally, there was a trend towards increased levels of IL-2, IL-6 and VEGF in GBM patients, which did not reach significance on a *p* < 0.01 level (U = 48.5, *p* = 0.023; U = 46, *p* = 0.017; U = 54, *p* = 0.043 respectively, Fig. 2C). In order to further characterize the cytokine pattern within the CSF we performed a principal component analysis (PCA). The PCA yielded an overall sampling adequacy of KMO = 0.783 with a KMO > 0.5 for the individual items and sufficiently large correlations as assessed by the Bartlett’s test of sphericity (Χ^2^(210) = 1127.002, *p* < 0.001). An initial analysis based on Kaiser’s criterion to extract factors with “eigenvalues” > 1 produced a five component solution and explained 93% of the variance. The screen plot, however, suggested a two component solution which still explained 80% of the total variance. Due to the relatively small sample size the analysis was continued with a two component solution. Since we expected correlations among the cytokines, we chose an oblique rotation method (Promax) and the components converged with a relatively large correlation of r = 0.605. The components matched well with the categories “chemokines” (component 1, including primarily MIP-1α, MIP-1β, eotaxin, eotaxin-3. IP-10, MDC, MCP-4, TARC, IL-8, IL-6 and IL-12p40) and “cytokine panel” and “proinflammatory panel” (component 2, including primarily IFN-γ, IL-4, IL-12p70, IL-2, IL-16, IL-13, IL-1β, IL-10 and TNF-α) of the original V-PLEX scales and were subsequently named “chemokines” and “inflammatory cluster”. Regression factor scores for each subject were saved and used in further analysis. There were no significant differences in the regression factor scores between GBM, AA and controls. These results indicate, that the cytokines within cluster component 1 (chemokines) and cluster component 2 (proinflammatory panel) are likely regulated in parallel.

### Correlations of immune cell infiltrates and cytokine concentrations in the CSF

We observed significant negative correlations between CD68^+^ macrophage infiltrations in the tumor tissue and the concentrations of the cytokines eotaxin, IFN-γ, IL-1β, IL-2, IL-10, IL-13, IL-16, VEGF and factor score of component 2 (“Inflammatory Cluster”) in the CSF. In addition, there was a trend for a positive correlation of IL-8 and IP-10/CXCL10 with perivascular CD163^+^ cell infiltrates as well as a trend for a positive correlation of the percentage of CD163^+^ cells and MDC-1/CCL22 in the tumor core.

**Fig. 3:**
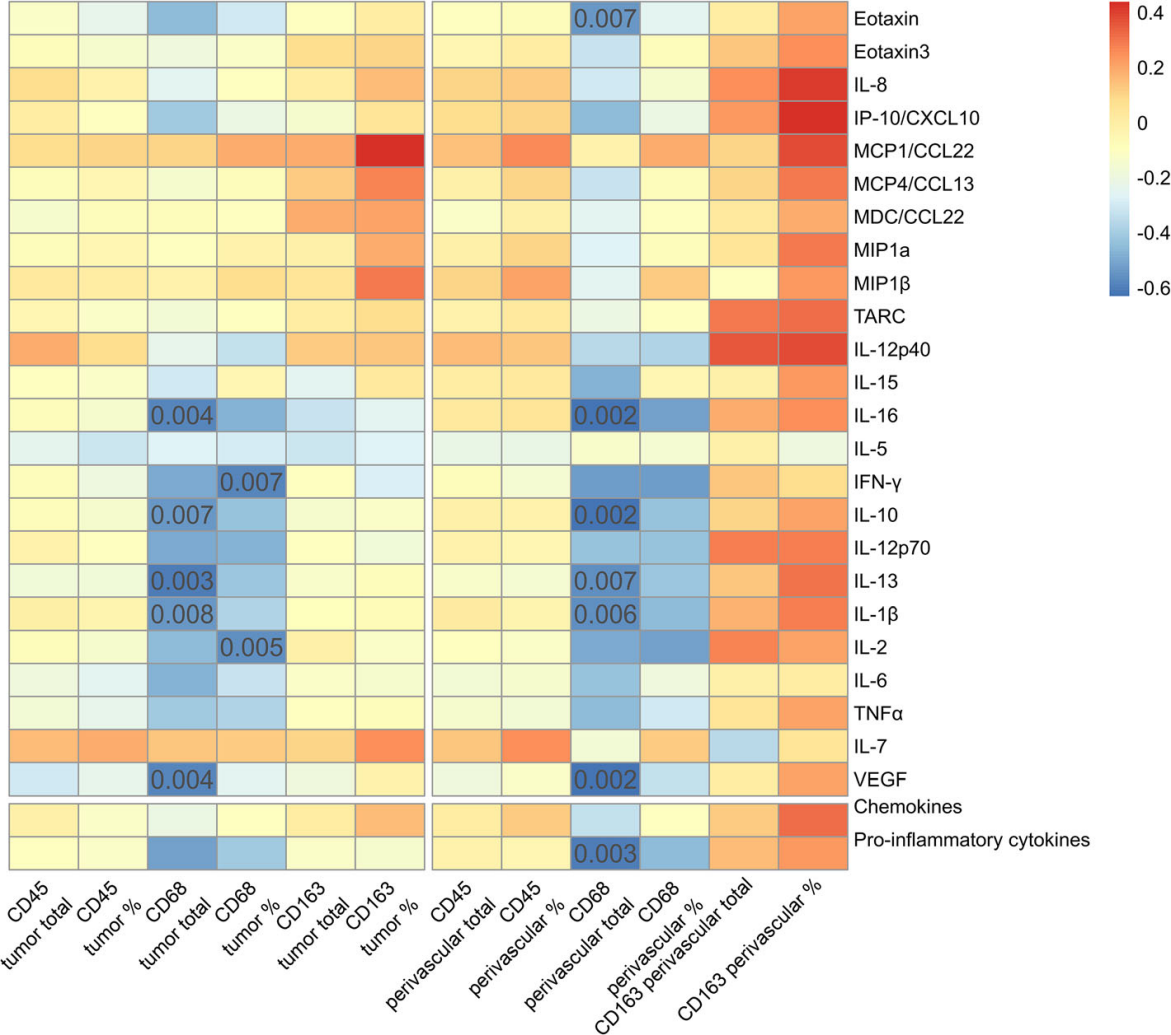
Correlations between cytokines and inflammatory cell infiltrates in the perivascular area. Correlations of the CSF cytokines and the factor regression scores with CD45, CD68 and CD163 with absolute cell counts and relative cell counts for the tumor core and perivascular tumor area are shown. Size and direction of the correlation are indicated by color as defined in the legend. Significant correlations on a *p* < 0.01 level are indicated by the *p* value in the matrix.

For a detailed overview on the correlations refer to Fig. 3 and supplementary Tables 2 and 3.

Fifty percent of the patients in the GBM group showed a disruption of the BBB. To assess whether this affected the results, we conducted a subgroup analysis testing for differences in cytokine concentration between these groups. We then reevaluated correlations across compartments for the subgroups. In general, there was no different effect of BBB disruption on the cytokine concentrations, but almost all correlations failed to reach significance in these subgroups. However, a deeper view into the correlation plots of both groups showed similar correlation patterns.

### Influence of MGMT promotor methylation status, sex, overall survival and age on immune cell infiltrations and cytokine concentrations

We observed no effect of MGMT promotor methylation on the cytokine concentrations, but a trend towards an increased percentage of CD163^+^ cells in the tumor tissue (U = 7.00, *p* = 0.025) for the patients presenting an unmethylated MGMT promotor region. However, this did not reach statistical significance on a *p* < 0.01 level. Further, there was no significant effect of sex on immune cell infiltration nor a significant correlation of age or overall survival with one of the variables (*p* > 0.01).

## Discussion

In this study, we assessed the interactions between immune cell infiltrates within different tumor areas and the corresponding cytokine concentrations in the CSF of patients diagnosed with HGG. Our findings on tissue resident immune cells suggest that lymphocyte numbers are generally low in HGG when compared to other brain tumor entities [27, 28]. However, in the panel of examined immune cells, macrophages represent the majority of infiltrating immune cells. Among the analyzed cytokines, IL-7 was significantly downregulated in HGG compared to controls whereas IL-2, IL-6 and VEGF showed a trend towards increased levels in the CSF of GBM patients compared to cytokine concentrations in age- and gender-matched controls. Regarding the correlation of immune cell infiltrates and cytokine concentrations in the CSF we found significant correlations between CD68^+^ macrophage infiltrates and eotaxin, IFN-γ, IL-1β, IL-2, IL-10, IL-13, IL-16 and VEGF. Given that the CSF has been considered a surrogate to assess pathophysiologic changes within the CNS [29], CSF cytokine levels may be associated with ongoing microenvironmental changes within the tumor and may possibly interfere with cell maturation, immune cell recruitment and infiltration into the CNS.

Overall, leucocyte infiltration in the surgically resected tumor tissue analyzed by CD45 staining was even lower than previously reported [30, 31]. Because of low cell counts, we did not attempt to further differentiate the phenotype of CD45+ lymphocytes into B or T cells. Additionally, the concentration of CXCL13, a predictor for B cell levels in the CSF [32] was below the detection limit in all samples. This data suggests that B cells or B cell follicular structures that occur during neuro-inflammatory processes and other tumor entities [29, 32–35] do not seem to play a major role in glioma immunity.

A higher degree of CD45+ cell infiltrate was found in the perivascular tumor area of GBM patients compared to the tumor core. This could be explained by the proximity to the peripheral blood from where lymphocytes and monocytes may cross the blood-brain-barrier [36]. Further, it has been shown in a glioma mouse model that brain microglia and macrophages accumulate in perivascular tumor areas and support tumor angiogenesis by the release of proangiogenic factors [37]. We also found a higher density of CD68^+^ macrophage infiltration in perivascular areas compared to the tumor core. However, the occurrence of M2-polarized macrophages in the perivascular space in our study was not as prominent as it has been described in a mouse model [37]. Instead, we found a tendency towards higher absolute macrophage infiltration (for both CD68^+^ and CD163^+^ cells) in the tumor core compared to the infiltration zone. Besides, we identified a tendency towards higher CD163+ M2 type macrophage infiltrations in WHO grade IV GBM compared to WHO grade III AA. Previous results support the notion of increasing macrophage infiltration from the periphery towards the tumor core [31].

When analyzing cytokine concentrations in the CSF, we detected a reduced concentration of IL-7 in the CSF of GBM patients compared to controls. IL-7 regulates T cell proliferation and survival [38, 39] and has been used in immunotherapeutic approaches for cancer therapies due to its immune reconstructive and activating properties [40]. Indeed, IL-7 in combination with IFN-γ significantly increased the survival time in an animal model for glioma [41]. In addition, we observed trends for elevated concentrations for the pro-inflammatory cytokines IL-2 and IL-6 and the angiogenic factor VEGF. An up-regulation of IL-6 in the CSF has been reported and associated with tumor infiltrating macrophages [19, 42]. Elevated VEGF concentrations in the CSF have repeatedly been found in previous studies [43–45]. Taken together, the relatively limited detection of CD45^+^ immune cells compared to the lymphocyte infiltration in other central nervous system tumors such as brain metastases [46, 47] and the slight changes in CSF cytokine profiles suggest, that the process of immunosurveillance within gliomas is limited when compared to other tumor entities.

Despite of the small changes in the CSF cytokine profile, a negative correlation was found between the number of CD68^+^ macrophages in the tumor tissue and the inflammatory cluster as revealed by our principal component analysis. In detail, the cytokines eotaxin, IFN-γ, IL-1β, IL-2, IL-10, IL-13, IL-16, and VEGF all showed a significant negative correlation with macrophage infiltration. Concerning the respective concentration changes in the CSF, a clear trend towards elevated CSF levels was observed for IL-2 and VEGF as described above, and slightly elevated levels were detectable for IL-1β, IL10 and IL13 whereas no changes were observed for the remaining cytokines (Fig. 2C, Suppl. Fig. 1).

Although cytokine functions cannot always be strictly assigned to a single distinct pathway, IL-2, IL-16 and IFN-γ have mostly been associated with pro-inflammatory states including e.g. the activation of T cells [18, 48, 49] and show multiple interactions with monocytes / macrophages. Monocytes can secrete IL-16 [50] and release inflammatory cytokines such as IL-1β, IL-5, IL-15 and TNF-α in response to IL-16 stimulation. However, this response is lost after differentiation of monocytes into mature macrophages [51]. In glioma patients, CD68^+^IL-16^+^ macrophages in the brain tissue have been identified as the major source of IL-16 expression correlating well with the glioma WHO grade and therefore its malignancy [52]. Among the pro-inflammatory cytokines, IFN-γ has been attributed a dual role in cancer immunology eliciting antitumor immunity on the one hand and inducing immune escape of tumor cells on the other [53]. In contrast, IL-10 has been clearly considered to serve as an immunosuppressive cytokine known to inhibit T cell activation, interfering with the IFN-γ induced activation of monocytes [54] and stimulating the conversion of monocytes towards pro-tumorigenic CD163+ M2 type macrophages [55]. Additionally, it was shown that IL-10 was released by M2 macrophages and promoted tumor proliferation in vitro [56].

Among the cytokines that correlate with CD68^+^ macrophages, IL-1β, IL-13 and VEGF have previously been described as tumor promoting factors. IL-1β is an inflammatory cytokine able to induce chronic inflammatory states by the activation of transcription pathways [57, 58]. In GBM it has been suggested that the IL-1β concentration is controlled in an autocrine loop by the expression of IL-1β, IL-1 receptors and IL-1 receptor antagonists [59]. IL-13 received attention as an important cytokine since a significant overexpression of the IL-13-receptor-α (IL-13Rα) has been detected in glioblastoma [60]. Consequently, personalized treatments with CAR-T cells targeting IL-13Rα have been employed with some promising outcomes [61]. Moreover, IL-13 is not only responsible for the conversion of macrophages towards the M2 phenotype [62] but also seems to play a role in the vascular cell adhesion molecule VCAM-1/Integrin α4β1 dependent infiltration of monocytes and macrophages [63]. Regarding VEGF, GBM has been associated with increased expression, subsequent dysregulation of angiogenesis leading to increased hypoxia [64]. Unfortunately, recurrence occurs despite anti-VEGF therapy and overall survival remains poor [64]. VEGF has immunosuppressive effects by e.g. recruiting tumor associated M2 type macrophages [65] and the increase in macrophages following antiangiogenic treatment has been proposed to play a critical role in evasion mechanisms of GBM and its therapy resistance [66, 67]. Further, the chemokine eotaxin showed elevated levels in GBM tumor tissue promoting tumor proliferation, migration and invasion, also showing an association with the overall patients’ survival [68]. In breast cancer models Tripathi et al. (2014) identified eotaxin as a key cytokine for macrophage migration and M2 type conversion [69]. The significant correlation of macrophages and eotaxin levels in the CSF found in our study suggest similar mechanisms in GBM patients.

Taken together, multiple cytokines that show a negative correlation with CD68^+^ macrophages have been associated with M2 phenotype macrophages or M2 type differentiation. However, when looking at CD163^+^ M2 type macrophages directly in our study, we found limited positive correlations with IL-8, IP-10 and MDC-1 which is in line with the literature as M2 type macrophages have been related to cytokine levels and cytokine dependent polarization [62, 70, 71].

Although the correlations between cytokines and immune cell subtypes identified in our study are supported by evidence from previous studies on cytokines and immune cells in GBM, causal relationships cannot be derived from our analysis. It might be speculated that a localized release in proximity to the tumor cells may result into a concentration gradient from the tissue to the CSF compartment leading to effects on macrophages. An alternative explanation for the negative correlations between inflammatory cytokines and macrophages could be that macrophages further differentiate within tumor tissues upon cytokine stimulation and undergo a change of surface marker expression. Indeed, we observed positive correlations between M2 type macrophages and a limited number of chemokines. However, to what extent cytokine levels in the CSF actually reflect microenvironmental changes within the tumor is worth to be discussed. Additional subanalyses in patients with and without a disrupted BBB showed that a disruption does not seem to influence cytokine concentrations in the CSF since we found the same trends in both subgroups. Moreover, the CSF / brain parenchyma barrier at the ependyma has generally been considered only a partial barrier [72]. Immunological interactions between the tumor tissue and CSF may therefore be less dependent on barrier disturbance. Interestingly, the cytokine expression profile in GBM does not seem to depend on the prognostically and therapeutically relevant MGMT promotor methylation. After all, the CSF may be considered to be a reliable surrogate to monitor microenvironmental changes in GBM.

Taken together, the role of cytokines during cancerogenesis and tumor progression remains ambiguous. On the one hand, immunosurveillance is supposed to suppress and control the development of abnormal cells and cancer, on the other hand, chronic inflammatory states are believed to promote cancer [4, 73]. Even though the mechanisms of glioma initiation are less clear, GBM eventually leads to a dysregulated immune response, which resembles a state of chronic inflammation including pro- and anti-inflammatory factors at the same time [74]. In GBM, like in other cancer types, this “immune chaos” favors tumor progression and invasiveness. Pro-inflammatory cytokines can induce cell survival, proliferation, migration and angiogenesis [57], while simultaneously a tumor environment is established which protects the tumor from the host’s immune response and surveillance by the release of immunoregulatory cytokines such as IL-10 [18]. Especially macrophages have been identified as key players in the dichotomous role of the immune response and their pro- and anti-inflammatory differentiation has been under intensive investigation [75]. Understanding and manipulating the cytokine profile of the tumor microenvironment is subsequently an interesting target for immunotherapy.

The following methodological limitations have to be considered in our study: I) the lack of IHC dual-staining due to the limited availability of tissue material did not allow for the differentiation between CD68^+^/CD163^−^ and CD68^+^/CD163^+^ cells, to assure that all CD163^+^ cells belonged to the macrophage population. Nevertheless, cell morphology suggested this assumption. Since the differentiation upon surface markers between the macrophage subsets is not very clear cut in the literature we used a more general staining of macrophages by using CD68 and only specified malignant M2 macrophages as previously described [22–24]. It will be interesting to more precisely describe the macrophage subsets that relate with CSF cytokines in future studies based on different mRNA / protein expression profiles. II) Small sample sizes and non-healthy control subjects had to be accepted due to few matched tissue and CSF samples for the disease group and few lumbar punctures in healthy subjects. In addition, longitudinal tissue samples for individual patients were not consistently available after radio- and chemotherapy which would have added interesting information on immunologic effects after therapy. III) Bias in the cytokine concentration measures could have resulted from the simultaneous analysis of 10 proteins per well which did not allow for optimal dilutions of every protein in the sample. However, all experiments were conducted according to the manufacturer’s instructions. IV) The overall survival for AA patients was below the survival for GBM patients, which is most likely explained by the small sample size, especially in the AA patient group.

## Conclusion

In summary, our results show that immune cell infiltration into gliomas is dominated by macrophages often representing the M2 phenotype. In contrast, CD45^+^ leukocytes were scarce in our analysis. The pro-inflammatory cytokine IL-7 was downregulated in HGG, pointing towards a possibly insufficient immunosurveillance by lymphocyte infiltrates within the tumor. Multiple correlations were observed between cytokine concentrations in the CSF and macrophages, suggesting that cytokines play a critical role in tumor pathogenesis and associated microenvironmental alterations. Considering the complex interaction patterns and interdependent regulation of pro- as well as non- and also anti-inflammatory cytokines, our data supports the notion of an immunological dysregulation in GBM. Targeting the cytokine profile in combination with other therapeutic approaches could possibly be utilized to affect the immune response in gliomas. However, more complex analyses including the cellular analysis of glioma cells and immune cells within the tumor tissue as well as CSF immune cells and cytokines in combination are necessary to further understand immunologic effects in gliomas.

## Supplementary Information

**Additional file 1: Table S1.** Data Cleaning. **Table S2.** Correlations between cytokines and inflammatory cell infiltrates in the tumor core. **Table S3.** Correlations between cytokines and inflammatory cell infiltrates in the perivascular area.

**Additional file 2: Fig. S1.** Trends in cytokine concentration differences. Cerebrospinal fluid concentrations in pg/ml of eotaxin, IFN-γ, IL-1β, IL-10, IL-13, IL16 and the regression factor score of the inflammatory cluster factor for glioblastoma patients and controls assessed by multiplex assay. Median values are presented for each group.

## Data Availability

Raw data were generated at the University of Tübingen. Derived data supporting the findings of this study are available from the corresponding author MK on request.

## Abbreviations

AA: Anaplastic astrocytoma
BBB: Blood brain barrier
CSF: Cerebrospinal fluid
GBM: Glioblastoma
GM-CSF: Granulocyte-macrophage colony-stimulating factor
HE: Hematoxylin/eosin
HGG: High grade glioma
HIF: Hypoxia-inducible factor
HIH: Hertie Institute for Clinical Brain Research
IDH: Isocitrate dehydrogenase
IFN: Interferon
IHC: Immunohistochemistry
IL: Interleukin
IL-13Rα: Interleukin-13 receptor α
LLoD: Lower limit of detection
MCP: Monocyte chemotactic protein
MGMT: O6-Methylguanin-DNA-Methyltransferase
MIP: Macrophage inflammatory protein
N.A.: Not available
OCB: Oligoclonal bands
PCA: Principal component analysis
TAM: Tumor associated macrophage
TARC: Thymus activation regulated chemokine
TNF: Tumor necrosis factor
ULoD: Upper limit of detection
VCAM − 1: Vascular cell adhesion molecule
VEGF: Vascular endothelial growth factor
WT: Wild type
