# Supplementary material for: Cerebrospinal fluid cytokine levels are associated with macrophage infiltration into tumor tissues of glioma patients
Source: BMC Cancer. 2021 Oct 15;21:1108. doi: 10.1186/s12885-021-08825-1 (PMC8520299; doi:10.1186/s12885-021-08825-1)
Supplement: Supplementary file 1 — Additional file 1: Table S1. Data Cleaning. Table S2. Correlations between cytokines and inflammatory cell infiltrates in the tumor core. Table S3. Correlations between cytokines and inflammatory cell infiltrates in the perivascular area. [file 12885_2021_8825_MOESM1_ESM.docx]

**Supplements**

**Table 1: Data Cleaning**

|  | **%Samples set to highest**  **Standard** | **% Samples set to 0** | **% of duplicates** |
| --- | --- | --- | --- |
| Eotaxin |  |  | 93.54 |
| Eotaxin-3 |  |  | 93.54 |
| IP-10 | 6.25 |  | 93.54 |
| MCP-1 | 31.25 |  | 93.54 |
| MCP-4 |  |  | 93.54 |
| MDC |  |  | 93.54 |
| MIP-1α |  |  | 93.54 |
| MIP-1β |  |  | 93.54 |
| TARC |  |  | 93.54 |
| M-CSF |  | 87.5 | 93.54 |
| IL-12p40 |  |  | 93.54 |
| IL-1α |  | 78.13 | 93.54 |
| IL-1β |  | 18.75 | 19.35 |
| IL-2 |  | 6.25 | 19.35 |
| IL-4 |  | 65.63 | 19.35 |
| IL-5 |  | 28.13 | 93.54 |
| IL-6 |  |  | 19.35 |
| IL-7 |  |  | 93.54 |
| IL-8 |  |  | 93.54 |
| IL-10 |  |  | 19.35 |
| IL-12p70 |  | 59.38 | 19.35 |
| IL-13 |  | 37.5 | 19.35 |
| IL-15 |  |  | 93.54 |
| IL-16 |  |  | 93.54 |
| IL-17 |  | 96.88 | 93.54 |
| TNF-α |  | 3.13 | 19.35 |
| TNF-β |  | 96.88 | 93.54 |
| VEGF | 3.125 | 3.13 | 93.54 |
| IFN-γ |  | 54.4 | 19.35 |
| CXCL13 |  | 100 | 100 |

***Table 1:*** Data cleaning of the cytokine concentrations for all subgroups. Percentages of the number of samples set to the highest standard which were exceeding the detection limit and percentage of samples set to zero which were below the detection limit are presented. Further, the percentage of samples measured in duplicates where enough cerebrospinal fluid material was available is shown.

**Table 2: Correlations between cytokines and inflammatory cell infiltrates in the tumor core**

|  | **CD45**  **tumor total** | **CD45**  **tumor %** | **CD68**  **tumor total** | **CD68**  **tumor %** | **CD163**  **tumor total** | **CD163**  **tumor %** |
| --- | --- | --- | --- | --- | --- | --- |
| Eotaxin | -0.12 | -0.23 | -0.45 | -0.30 | -0.10 | 0.01 |
| Eotaxin3 | -0.08 | -0.14 | -0.19 | -0.12 | 0.08 | 0.10 |
| IL-8 | 0.08 | -0.03 | -0.25 | -0.10 | 0.01 | 0.16 |
| IP-10/CXCL10 | 0.01 | -0.10 | -0.41 | -0.21 | -0.14 | 0.05 |
| MCP1/CCL22 | 0.08 | 0.10 | 0.10 | 0.19 | 0.19 | 0.44 |
| MCP4/CCL13 | -0.08 | -0.05 | -0.14 | -0.08 | 0.12 | 0.27 |
| MDC/CCL22 | -0.14 | -0.08 | -0.08 | -0.10 | 0.19 | 0.21 |
| MIP1a | -0.08 | -0.05 | -0.10 | -0.03 | -0.01 | 0.19 |
| MIP1β | 0.03 | 0.01 | -0.03 | 0.08 | 0.05 | 0.30 |
| TARC | -0.05 | -0.12 | -0.16 | -0.10 | 0.01 | 0.08 |
| IL-12p40 | 0.19 | 0.08 | -0.23 | -0.34 | 0.12 | 0.14 |
| IL-15 | -0.10 | -0.12 | -0.30 | -0.05 | -0.25 | 0.03 |
| IL-16 | -0.08 | -0.14 | **-0.58*** | -0.47 | -0.32 | -0.25 |
| IL-5 | -0.24 | -0.31 | -0.27 | -0.29 | -0.31 | -0.27 |
| IFN-γ | -0.08 | -0.20 | -0.50 | **-0.58*** | -0.10 | -0.28 |
| IL-10 | -0.08 | -0.14 | **-0.54*** | -0.43 | -0.14 | -0.12 |
| IL-12p70 | -0.03 | -0.10 | -0.50 | -0.48 | -0.10 | -0.18 |
| IL-13 | -0.17 | -0.17 | **-0.60*** | -0.42 | -0.13 | -0.08 |
| IL-1β | 0.00 | -0.04 | **-0.54*** | -0.38 | -0.09 | -0.07 |
| IL-2 | -0.08 | -0.14 | -0.45 | **-0.56*** | -0.01 | -0.12 |
| IL-6 | -0.19 | -0.25 | -0.47 | -0.32 | -0.12 | -0.14 |
| TNFα | -0.16 | -0.23 | -0.41 | -0.38 | -0.10 | -0.08 |
| IL-7 | 0.16 | 0.19 | 0.14 | 0.12 | 0.10 | 0.25 |
| VEGF | -0.30 | -0.23 | **-0.58*** | -0.25 | -0.19 | -0.03 |
| Chemokines | -0.01 | -0.12 | -0.21 | -0.10 | 0.01 | 0.16 |
| Pro-inflammatory cytokines | -0.10 | -0.12 | -0.52 | -0.41 | -0.12 | -0.14 |

***Table 2****:* Correlations of the CSF cytokines and the factor regression scores for CD45, CD68 and CD163 with absolute cell counts and relative cell count for core tumor tissue. Significant correlations on a p<0.01 level are indicated by an asterisk (*).

**Table 3: Correlations between cytokines and inflammatory cell infiltrates in the perivascular area**

|  | **CD45**  **perivascular total** | **CD45**  **perivascular %** | **CD68**  **perivascular total** | **CD68**  **perivascular %** | **CD163 perivascular total** | **CD163 perivascular %** |
| --- | --- | --- | --- | --- | --- | --- |
| Eotaxin | -0.10 | -0.08 | **-0.54*** | -0.25 | 0.01 | 0.21 |
| Eotaxin3 | -0.05 | 0.01 | -0.32 | -0.08 | 0.14 | 0.25 |
| IL-8 | 0.10 | 0.12 | -0.30 | -0.14 | 0.25 | 0.41 |
| IP-10/CXCL10 | 0.08 | 0.10 | -0.45 | -0.21 | 0.23 | 0.43 |
| MCP1/CCL22 | 0.15 | 0.26 | -0.03 | 0.19 | 0.10 | 0.38 |
| MCP4/CCL13 | -0.01 | 0.10 | -0.32 | -0.08 | 0.10 | 0.30 |
| MDC/CCL22 | -0.12 | -0.01 | -0.25 | -0.10 | 0.03 | 0.19 |
| MIP1a | -0.01 | 0.10 | -0.27 | -0.08 | 0.05 | 0.30 |
| MIP1β | 0.10 | 0.21 | -0.25 | 0.12 | -0.10 | 0.23 |
| TARC | -0.03 | 0.03 | -0.21 | -0.10 | 0.30 | 0.32 |
| IL-12p40 | 0.16 | 0.14 | -0.36 | -0.38 | 0.36 | 0.38 |
| IL-15 | 0.01 | 0.03 | -0.47 | -0.05 | -0.01 | 0.23 |
| IL-16 | 0.03 | 0.05 | **-0.63*** | -0.52 | 0.19 | 0.25 |
| IL-5 | -0.22 | -0.22 | -0.13 | -0.15 | -0.02 | -0.20 |
| IFN-γ | -0.08 | -0.15 | -0.53 | -0.53 | 0.13 | 0.08 |
| IL-10 | -0.01 | -0.03 | **-0.63*** | -0.43 | 0.10 | 0.21 |
| IL-12p70 | -0.03 | -0.05 | -0.43 | -0.43 | 0.28 | 0.28 |
| IL-13 | -0.13 | -0.15 | **-0.56*** | -0.42 | 0.13 | 0.31 |
| IL-1β | 0.02 | -0.04 | **-0.56*** | -0.45 | 0.18 | 0.29 |
| IL-2 | -0.10 | -0.12 | -0.50 | -0.52 | 0.27 | 0.21 |
| IL-6 | -0.16 | -0.14 | -0.43 | -0.19 | -0.01 | 0.01 |
| TNFα | -0.14 | -0.16 | -0.45 | -0.30 | 0.05 | 0.21 |
| IL-7 | 0.14 | 0.25 | -0.16 | 0.12 | -0.36 | 0.05 |
| VEGF | -0.19 | -0.12 | **-0.63*** | -0.34 | 0.01 | 0.21 |
| Chemokines | 0.01 | 0.12 | -0.34 | -0.10 | 0.12 | 0.32 |
| Pro-inflammatory cytokines | -0.03 | -0.05 | **-0.60*** | -0.45 | 0.16 | 0.23 |

***Table 3****:* Correlations of the CSF cytokines and the factor regression scores for CD45, CD68 and CD163 with absolute cell counts and relative cell count for the perivascular tumor area. Significant correlations on a p<0.01 level are indicated by an asterisk (*).
